# Vibrational Spectroscopy: A Valuable Screening and Diagnostic Tool for Obstetric Disorders?

**DOI:** 10.3389/fgwh.2020.610582

**Published:** 2021-01-28

**Authors:** Oliver Richards, Cerys Jenkins, Helena Griffiths, Edyta Paczkowska, Peter R. Dunstan, Sharon Jones, Margery Morgan, Tanya Thomas, Jayne Bowden, Annettee Nakimuli, Manju Nair, Catherine A. Thornton

**Affiliations:** ^1^Institute of Life Science, Swansea University Medical School, Swansea University, Swansea, United Kingdom; ^2^Department of Physics, College of Science, Swansea University, Swansea, United Kingdom; ^3^Maternity and Child Health, Singleton Hospital, Swansea Bay University Health Board, Swansea, United Kingdom; ^4^Department of Obstetrics and Gynaecology, School of Medicine, Makerere University College of Health Sciences, Kampala, Uganda

**Keywords:** preeclampsia, gestational diabetes, vibrational spectroscopy, Raman spectroscopy, fourier-transform infrared spectroscopy (FTIR), screening, diagnosis

## Abstract

Preeclampsia (PE) is a common obstetric disorder typically affecting 2–8% of all pregnancies and can lead to several adverse obstetric outcomes for both mother and fetus with the greatest burden of severe outcomes in low middle-income countries (LMICs), therefore, screening for PE is vital. Globally, screening is based on maternal characteristics and medical history which are nonspecific for the disorder. In 2004, the World Health Organization acknowledged that no clinically useful test was able to predict the onset of PE, which prompted a universal search for alternative means of screening. Over the past decade or so, emphasis has been placed on the use of maternal characteristics in conjunction with biomarkers of disease combined into predictive algorithms, however these are yet to transition into the clinic and are cost prohibitive in LMICs. As a result, the screening paradigm for PE remains unchanged. It is evident that novel approaches are needed. Vibrational spectroscopy, specifically Raman spectroscopy and Fourier-transform infrared spectroscopy (FTIR), could provide better alternatives suited for implementation in low resource settings as no specialized reagents are required for conventional approaches and there is a drive to portable platforms usable in both urban and rual community settings. These techniques are based on light scattering and absorption, respectively, allowing detailed molecular analysis of samples to produce a unique molecular fingerprint of diseased states. The specificity of vibrational spectroscopy might well make it suited for application in other obstetric disorders such as gestational diabetes mellitus and obstetric cholestasis. In this review, we summarize current approaches sought as alternatives to current screening methodologies and introduce how vibrational spectroscopy could offer superior screening and diagnostic paradigms in obstetric care. Additionally, we propose a real benefit of such tools in LMICs where limited resources battle the higher prevalence of obstetric disorders.

## Introduction

Antenatal screening programmes are used around the world to identify pregnant women at risk of common obstetric disorders. These programmes result in improved maternal and fetal outcomes and identify those at need of additional antenatal care. Common screening procedures involve maternal biochemical tests for infections, screening for fetal syndromes such as Down's syndrome, and fetal morphological examinations for structural anomalies which are sensitive and specific. In contrast, several screening paradigms used for pregnancy complications such as preeclampsia (PE) and gestational diabetes mellitus (GDM) commonly rely upon risk factor based screening at booking appointments, familial medical history and the development of symptoms prior to diagnosis. The current screening procedure for PE is limited to assessing maternal characteristics to determine the risk of later PE development, while blood pressure and proteinuria estimations are used to confer a diagnosis of PE. These measures are non-specific and a focus on developing superior screening and diagnostic tests for PE has emerged. These new tests are based on combining maternal characteristics and biophysical measures into diagnostic and screening algorithms. Although progress has been made, clinical translation of these tests remains limited due to the variable performance in different patient populations and the unknown cost-effectiveness of introducing these approaches (1), both of which are included in the World Health Organisation (WHO) principles of screening (2).

Universal demand for improved screening and diagnostic tests for many types of non-obstetric diseases and disorders has recently geared toward the use of vibrational spectroscopy. Combining vibrational spectroscopy with algorithms offers the possibility of simple and automated disease classification tools suitable in a clinical setting. In vibrational spectroscopy, chemical bonds within molecules differentially interact with incident light, yielding valuable information about the nature and composition of the sample of interest generating a unique spectral fingerprint suitable for diagnostic use and that can be applied to the types of samples routinely collected for diagnostics especially plasma, serum and urine. Additionally, vibrational spectroscopy can support the identification of novel disease biomarkers to assist in future test design.

This review will summarize how vibrational spectroscopy might provide a novel screening and diagnostic tool for obstetric diseases with a focus on PE. Therefore, the screening and diagnostic paradigms of PE will be outlined along with consideration of their limitations and recent progress in developing superior tests.

## Current Screening and Diagnosis of Preeclampsia

Hypertensive disorders, including preeclampsia, chronic hypertension and gestational hypertension, are the second most common cause of maternal mortality worldwide accounting for 14% of all maternal deaths annually (3). PE typically affects around 4.6% of pregnancies globally, with considerable variation between countries (4). Two different subtypes of PE exist: early- (<34 weeks) and late- (>34 weeks) onset PE. Both can lead to severe maternal morbidity, mortality and fetal complications, with early-onset classed as the more severe subtype leading to higher risks of adverse obstetric outcomes (5) and more likely to recur. A failure in diagnosis or monitoring of PE can lead to more severe conditions such as eclampsia distinguished by the onset of seizures, and the syndrome of Haemolysis, Elevated Liver Enzymes, and Low Platelets (HELLP syndrome). Furthermore, PE can precede other maternal conditions such as abruptio placentae, pulmonary oedema and aspiration pneumonia, and fetal complications including intrauterine growth restriction (6–8). Although the exact aetiology is unknown, sufficient evidence suggests that impaired placentation in the first trimester and subsequent maternal angiogenic syndrome in the second and third trimesters contribute to the development of PE (9, 10).

Early recognition of PE is one of the major challenges in obstetric care worldwide. Globally, screening paradigms involve assessing women for risk factors for the development of PE and subsequent monitoring of disease development. Current PE screening guidelines for the UK issued by the National Institute for Health and Care Excellence (NICE) involve determining a women's risk at the booking appointment during the first trimester. These include: >40 years of age, nulliparity, a 10-year pregnancy interval, a previous diagnosis of preeclampsia or family history of the disease, BMI > 30, pre-existing vascular or renal disease and multiple pregnancies (11). Women deemed at risk of PE are recommended to have frequent blood pressure measurements and urinalysis for proteinuria throughout their antenatal care. Women with a diastolic reading of 110 mmHg or two consecutive readings of 90 mmHg at least 4 h apart and/or significant dipstick proteinuria test (+1) should have increased monitoring. If a positive dipstick reading (+1 or higher) is obtained, the albumin:creatinine or protein:creatinine ratios should be quantified with thresholds of 30 and 8 mg/mmol, respectively, indicating significant proteinuria. Treatment for preeclampsia should be considered if the systolic reading is above 160 mmHg on two consecutive readings at least 4 h apart. This care pathway is common around the world with slight modifications. For example, guidelines from the American College of Obstetricians and Gynaecologists (ACOG) resemble the NICE guidelines with minor modifications in blood pressure and proteinuria quantification values. In Uganda, like most of Sub Saharan Africa (SSA), screening relies mainly on assessing risk factors in the mothers, blood pressure measurement, and dipstick proteinuria; limited resources make further laboratory tests unfeasible for the majority of women.

## Limitations of Current Screening and Diagnosis

Assessing individual maternal characteristics and medical history to screen for PE is simple and of minimal cost, although its predictive value has been subject to much debate. A previous history of preeclampsia confers the highest predictive risk factor with a relative risk value of over 7 (12). However, this is of no value when determining the risk of nulliparous women and only just over half of women who later develop early- and late-term PE are detected using current NICE guidelines (13). Current NICE guidelines are reasonably sensitive as they can predict 77% (95% CI: 65.0–87.0) of PE cases at booking appointment, however, this comes with a significantly low degree of specificity at 54% (95% CI: 44.0–64.0) with a positive predictive value of only 7% based on a 4% incidence of PE (14). In addition to the discussion concerning risk-based screening for PE, concerns have also emerged regarding the diagnostic criteria and monitoring procedures. Blood pressure measurements during pregnancy are essential to clinical decision making around diagnosis and monitoring of PE, however methodological inaccuracies and user variability, along with variability between devices may affect their diagnostic performance (15, 16). Methods to assess proteinuria, the other main distinguishing feature of PE, also vary. The gold standard method to quantify proteinuria is the 24-h urine collection test, which is costly, time-consuming and impractical. Consequently, alternative methods have been introduced such as the protein:creatinine and albumin:creatinine ratios and the semi-quantitative urine dipstick test which are simple and economical to perform. However, a high degree of variation in the protein:creatinine ratio suggests that this test is more reasonably a method to “rule-out” the presence of proteinuria instead of accurately diagnose it (17). There are also suggestions that proteinuria, regardless of means of testing, has limited clinical value and using such a test to inform clinical decisions is inappropriate as proteinuria is a poor predictor of maternal and fetal complications (18).

## Recent Advances in Novel Predictive Tests

PE prognosis relies on early recognition, timely diagnosis and increased monitoring to reduce the risk of maternal and fetal morbidity and mortality. Therefore, early screening and subsequent accurate diagnosis of these conditions are of utmost importance to identify those at risk of disease development and such paradigms have been investigated thoroughly and subjected to much debate. It is evident that alternative screening and diagnostic tests for PE which are sensitive, specific and simple to administer are needed, and much attention has focused on such necessity over the past decade. Advances in treatment and interventions also highlight the need for better screening and diagnostic strategies and tools. For example, the ASPRE study suggested that early prophylactic use of aspirin during gestation (150 mg/day) significantly reduced the incidence of preterm PE by 62% (19). Meta-analysis highlighted that to reduce the incidence of preterm PE, aspirin intervention must commence before 16 weeks of gestation (20). Therefore, early recognition is paramount to reducing maternal and fetal morbidity and mortality for early-onset PE. In 2004, a systemic review of screening tests for PE by the WHO concluded that no useful clinical screening test was presently available to predict the onset of PE (21). Subsequently, a considerable number of studies have explored alternative screening methodologies for predicting PE, involving biophysical and biochemical factors as singular predictive factors or in combination in predictive algorithms.

### Mean Arterial Pressure and Uterine Artery Doppler

A major hallmark of PE is new-onset hypertension. In a cohort of women who later developed PE, pre-gravid and first trimester mean arterial pressure (MAP) was significantly higher than in those women who did not develop PE (22). Subsequently, the discriminating power of MAP to screen for PE was investigated. However, although increased MAP is deemed a risk for the development of PE its independent predictive performance is limited. Respective studies have shown that MAP (cut off of >92 mm Hg) before 20 weeks of gestation and between 11.0 and 13^+6^ weeks has poor detection rates for PE at 25 and 37.5%, respectively, for a false positive rate of 10% (23, 24).

Improper trophoblast invasion is one of the main etiological components of PE and results in defective uteroplacental circulation measurable as increased pulsatility index and resistance in uterine arteries (25). Uterine artery blood flow can be measured trans-abdominally or trans-vaginally by Doppler ultrasound (26). A large scale metanalysis of 18 studies and over 55,000 women indicated that the Doppler ultrasound for the prediction of early-onset PE and both early- and late-onset PE was highly specific at 92.1% (95% CI: 88.6–94.6) and 93.4 (95% CI: 90.4–95.5), respectively. However, this was accompanied by poor predictive sensitivity; for early-onset PE (47.8%; 95% CI: 39.0–56.8) and for early- and late-onset PE (26.4%; 95% CI: 22.5–30.8) (27).

### Biomarkers

Discriminatory biomarkers are highly desirable in diagnostics and subsequent management of many diseases. The rise of “OMIC” based approaches particularly with respect to metabolomics and proteomics has resulted in the identification of an abundance of biomarkers for various disease states over the past few decades (28). Most candidate biomarkers for PE are of placental origin or involved in angiogenesis. Decreased levels of pregnancy associated plasma protein A (PAPP-A), involved in trophoblast invasion, is associated with increased risk of PE development (29, 30). Similarly, altered levels of placental growth factor (PlGF) and its target soluble fms-like tyrosine kinase-1 (sFlt-1), both involved in remodeling of uterine arteries, are observed as the sFlt-1/PlGF ratio in the first trimester which is significantly lower in women who later develop PE (31). However, in the weeks before PE onset, it appears that levels of sFlt-1 increase accompanied by decreasing levels of PlGF (30). As a result, NICE currently recommends PlGF testing between 20 and 36^+6^ weeks to aid the diagnosis of PE and a recent multicentre study suggests such testing reduces both the time taken for clinicians to diagnose PE and the incidence of adverse obstetric outcomes (32, 33). Despite altered levels of PlGF and sFLT-1 in women who develop PE, their performance in predicting later development of PE is inadequate prior to 20 weeks of gestation (34, 35). Also, the utilization of these biomarkers alone as independent early predictors of PE development is prevented as levels of these factors differ significantly from 19 weeks of gestation onwards (36). Furthermore, no single, specific biomarker exists for PE and the use of biomarkers independently in early pregnancy is discounted as a considerable crossover of these non-specific biomarkers exists in different disease states and conditions such as cardiovascular disease and Down's syndrome (37–39).

### Multivariate Approaches

From the above, standalone biochemical and biophysical parameters lack specificity and sensitivity for the prediction of PE. An early study showed that combining maternal characteristics into an algorithm rather than treating each factor individually improves detection rates: a false positive rate of 64.1% with detection rates of 89.2 and 93% for early and late-onset PE using NICE guidelines but a 5% false-positive rate and detection rates of 37.0 and 28.9% on combining maternal characteristics (40). Consequently, to improve detection rates, the emphasis has shifted to applying multivariate approaches to combine biochemical, biophysical and maternal characteristics into risk algorithms for the prediction of PE development. Combining maternal characteristics with biophysical parameters including Doppler ultrasound measurements and MAP, and biochemical markers of placental dysfunction including PAPP-A and PlGF into a predictive algorithm significantly improves the sensitivity, in particular for early-onset PE to 93.1% with a 5% false-positive rate (41). Numerous studies have followed this approach with varying detection rates for PE but external validation of the algorithms in different patient populations have failed to replicate comparable detection rates (42). More recently, a Bayes theorem based algorithm was developed (43). A validation study issued by The National Institute for Health Research (NIHR; UK) revealed a detection rate of 82.4% which was far superior to the detection rate of 41.6% using NICE guidelines at the same screen positive rate (44). Despite the superiority of multivariate approaches as opposed to current screening procedures for PE, they are yet to transition into clinical use as they are yet to abide by the WHO principles of screening.

Undoubtedly, by combining several parameters a significant improvement in the prediction of PE will be achieved. As PE is a multisystem disorder of heterogeneous etiology, a more comprehensive, global analysis to identify those at risk is required. Vibrational spectroscopy could offer an avenue to improve recent progress in the development of enhanced screening paradigms and might provide an alternative diagnostic approach especially if combined with risk factors and other clinical metadata. Recently, incorporating patient characteristics including age, gender and skin type to the diagnosis of several types of skin cancer using one type of vibrational spectroscopy known as Raman spectroscopy significantly improved sensitivity and specificity (45). The principle, current applications, and potential value of vibrational spectroscopy in obstetric care are discussed below.

## Principle of Vibrational Spectroscopy

**Figure 1:**
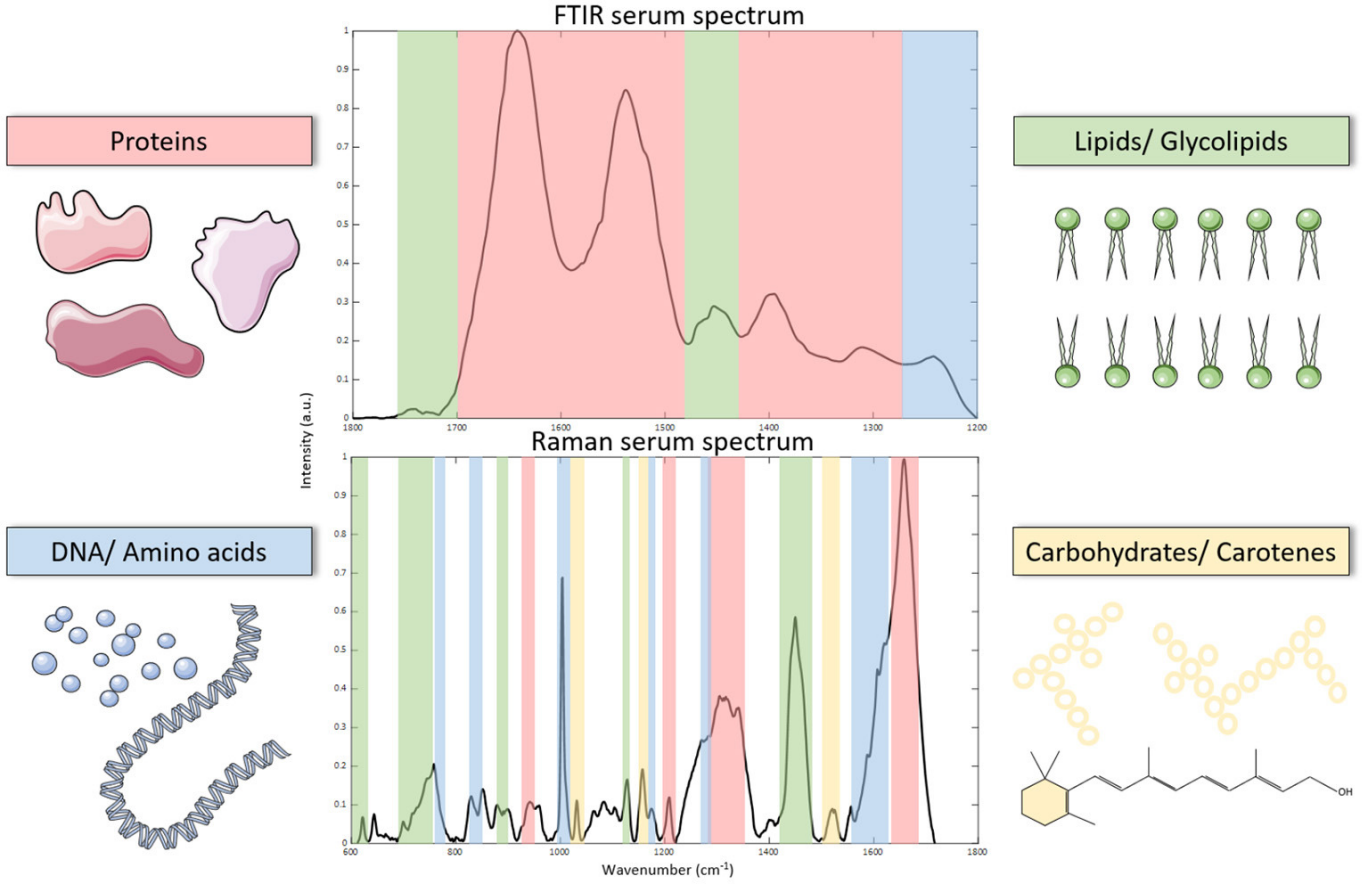
Processed FTIR and Raman spectroscopy spectrum of serum (fingerprint regions). Spectra can be used to identify biomolecules which correspond to their vibrational modes as illustrated on each spectrum.

Vibrational spectroscopy is a branch of spectroscopy which enables sensitive analysis of vibrational modes within a molecule or material. Molecules within a sample can have different vibrational modes with specific vibrational frequencies which are characteristic of their molecular structure and biological composition. Probing these molecular vibrations using an incident light source can relay valuable information about the composition of samples. For example, the presence of carbohydrates, proteins and lipids in a liquid biopsy such as serum or plasma, will yield a spectral output which represents a unique molecular fingerprint of the sample (Figure 1).

**Figure 2:**
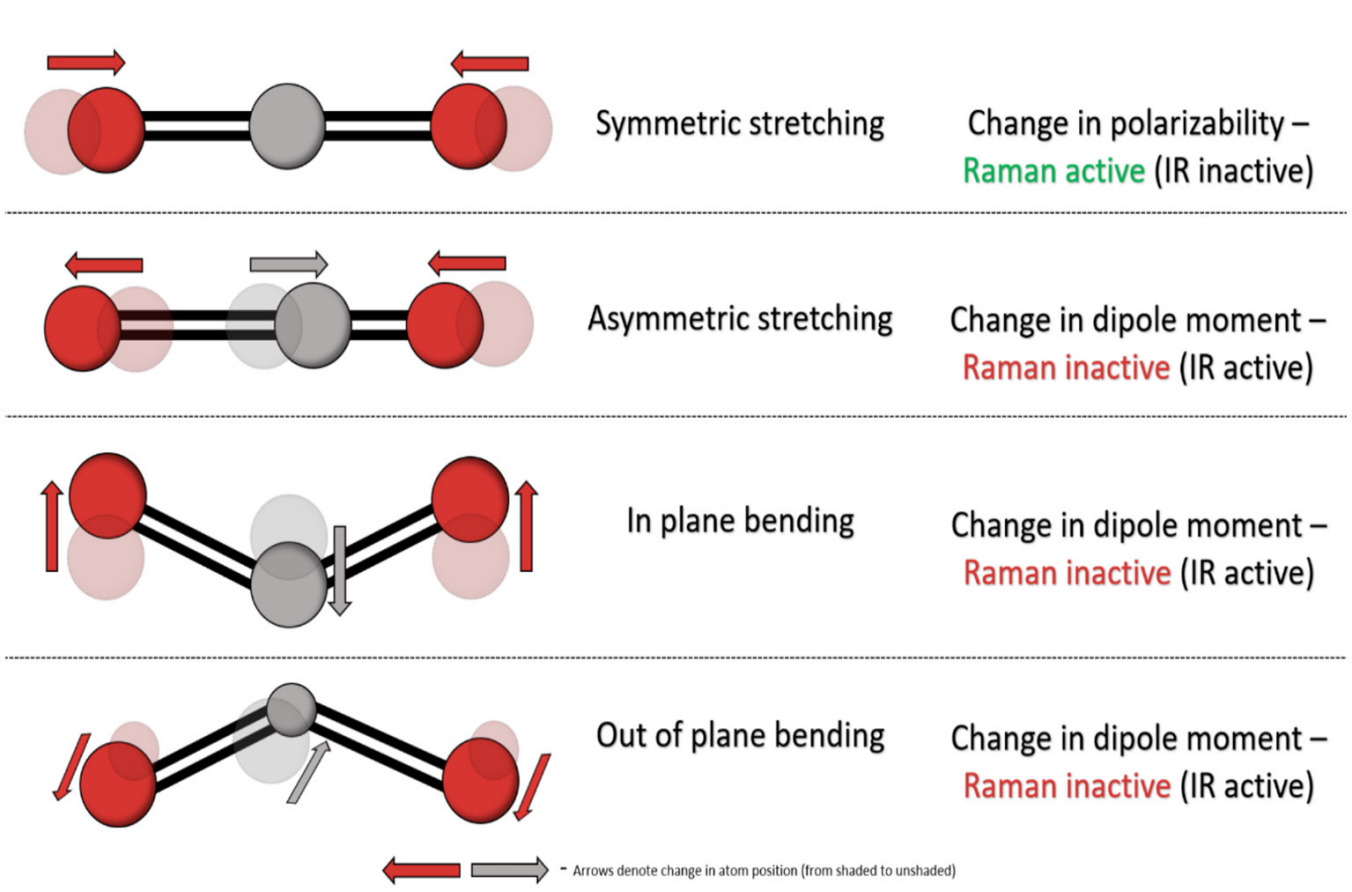
Vibrational modes of CO_2_ as an example and their relative FTIR and Raman activity.

Fourier-transform infrared (FTIR) and Raman spectroscopy are common and complementary vibrational spectroscopic techniques which provide information on the vibration modes present within a material. However, fundamentally and experimentally they differ significantly and they have distinct gross selection rules. FTIR spectroscopy involves the absorption of infrared light resulting in excitation of a molecule from its ground vibrational state to an excited vibrational state, altering its bond properties and causing a change in dipole moment—the charge distribution of molecular bonds. In contrast, Raman spectroscopy relies upon the relatively weak phenomenon called inelastic scattering where scattered photons are of different energy to the incident photons. Such scattering occurs as a result of induced molecular dipoles in polarisable bonds where their electron cloud can be disrupted as a result of vibrational transitions within molecules. Each molecule contains a set number of vibrational modes, and a non-linear molecule contains 3N-6 (*N* = total number of atoms) vibrational modes with distinct FTIR and Raman activity as illustrated in Figure 2.

Although traditionally used to probe chemical and physical properties of materials, there has been a notable rise in using both techniques in several fields of biological research including botany (46), microbiology (47, 48) and pharmacology (49, 50). In the past decade, a focus on the use of FTIR and Raman in disease discrimination and diagnostics has been established (51). Any given disease is associated with a change in biochemistry as a causative factor of the disease or as a result of disease manifestation, and vibrational spectroscopy can discriminate between multiple disease states. Vibrational spectroscopy unveils a wealth of biochemical information and is highly sensitive to minute changes in the chemical composition of samples, allowing discrimination between healthy and diseased states.

## Application of Raman Spectroscopy in Disease Diagnostics

The rise of vibrational spectroscopy in diagnostics is credited to technological advancements which have maximized the sensitivity of instruments. Raman scattering is a relatively weak phenomenon that has been overcome by advances such as the ability to use a range of laser excitation wavelengths, combined with increasingly sensitive detectors, thereby examining a wider potential of biomarkers from the sample within one instrument. Similar factors have advanced FTIR where for example using array detectors has facilitated large scale mapping capabilities. Additionally, enhanced computational and data mining processing has enabled rapid, user-friendly analysis of large spectral data sets. Spectral data includes in excess of a thousand variables which include wavenumbers and their relative intensities (52). Chemometric approaches are sought due to the complexity and diverse nature of biological spectra.

Vibrational spectroscopy possesses several advantages over traditional diagnostic and screening procedures. Experimental procedures for both FTIR and Raman spectroscopy allow biological samples to be analyzed at high-throughput capacities without causing significant damage to the sample. Water produces a relatively weak contribution to Raman spectra, so Raman spectroscopic analysis is highly compatible with biological samples such as plasma and serum. While aqueous solutions are not compatible with FTIR due to the strong absorption property of polar OH groups samples can be analyzed in their dried state. Consequently, sample preparation is minimal for both Raman spectroscopy and FTIR, which is desirable in any clinical setting with no added requirements of specialized reagents for conventional vibrational spectroscopic techniques. If required, more sophisticated and sensitive variations of vibrational spectroscopy are available. For example, surface-enhanced Raman spectroscopy is a powerful approach that uses electromagnetic field enhancement to enhance Raman scattering (53).

Most attention on vibrational spectroscopy in health and disease has been toward application for the leading causes of global mortality namely infectious diseases, cancer and cardiovascular diseases (54). In 2018, for example, an estimated 9.1 million deaths were attributed to cancer and its incidence and mortality are increasing globally (55). Owing to this, it is no surprise that the application of vibrational spectroscopy in cancer diagnostics has been a collective ambition of bio-spectroscopists over the past decade. Diagnosis is crucial to the prognosis of cancer and improving current diagnostic paradigms is a common goal amongst clinicians and scientists. These combined efforts involve developing real-time diagnostic technologies which are sensitive, specific and are non-invasive for patients. The use of body fluids for medical diagnostics is highly attractive to clinical services due to the minimal invasiveness compared to traditional biopsies. Plasma and serum are of considerable interest concerning their diagnostic potential. Plasma and serum harbor thousands of analytes including proteins, carbohydrates and lipids which arise from cells and tissues (56) and preparation of these bodily fluids is achieved relatively simply. To date, both FTIR and Raman spectroscopy of serum has shown promise as a technique for the diagnosis of various cancers including brain, breast, prostate, cervical, colorectal and lung cancer (57–59). Moreover, in addition to its predictive capability, Raman spectroscopy can also determine stages of cancer malignancy (60). Vibrational spectroscopy has shown promise in conditions other than cancer. Raman spectroscopy of serum can successfully identify patients with inflammatory conditions such as asthma (61), sepsis and systemic inflammatory response syndrome (62). The potential of blood-based vibrational spectroscopy in the diagnosis of neurodegenerative diseases such as Alzheimer's and Parkinson's has been demonstrated (63–66). Additionally, other sample types such as saliva and urine have shown diagnostic capabilities in other disease types such as gastric cancer (67) and oral disease (68).

## Vibrational Spectroscopy and Pregnancy

Given the extent of testing that occurs during pregnancy, vibrational spectroscopy might prove an incredibly useful tool given its ease of use and multiplex outputs. However, even preliminary testing of its potential is relatively sparse in obstetrics. Early studies involved the use of Raman spectroscopy in assisted reproductive technology to predict the viability of embryos used in *in vitro* fertilization. Profiling embryo culture media following implantation allowed prediction of embryo viability which later correlated to pregnancy outcomes (69, 70). FTIR has been similarly investigated attaining sensitivity and specificities of over 90% in determining a viable pregnancy (71). Raman spectroscopy has been used to detect changes in cervix composition *in vivo* in mice during normal pregnancy and delayed parturition (72, 73). The use of Raman spectroscopy in understanding biochemical cervical changes has also translated into humans revealing that cervical extracellular matrix proteins (at wavenumbers 1,248 and 1,254 cm^−1^) decrease throughout gestation, while unidentified blood components (at 1,233 and 1,563 cm^−1^) increase (74). Recently, a visually guided Raman spectroscopy probe which integrates into standard prenatal care has been developed for the measurement of biochemical changes of the cervix for the prediction of preterm birth, which may assist in the translation of such a device into obstetric care (75). As the search for alternative and novel screening methods for PE continues, the possibility that vibrational spectroscopy might address these needs has been considered.

## Discussion

On the basis that vibrational spectroscopy can accurately discriminate multiple disease and healthy states, these technologies could be applied more widely to obstetric care fulfilling the need for alternative screening and diagnostic technologies. The utility of vibrational spectroscopy for providing quantitative analysis of urine to inform PE diagnosis has been considered. Numerous studies have pursued the quantitative analysis of clinically relevant concentrations of uric acid in the urine of women with PE using surface-enhanced Raman spectroscopy but direct screening capabilities are yet to be determined (76–78). A preliminary study demonstrated that FTIR spectral differences between normotensive and women who later developed PE were characteristic of alterations in protein secondary structure and lipid profiles. While the authors claimed that using the amide A:amide B ratio accurately facilitated the diagnosis of PE after the 20th week of gestation with 92.9% confidence limits this was a very small study of 14 women that did not use a multivariate analysis approach (79). In another study, metabolic profiling of maternal serum using FTIR in early-onset and late-onset PE revealed 13 significant spectral peak differences between the two subject groups. Following discriminant analysis, five highly significant peaks (1,078, 1,088, 1,122, 1,169, and 1,171 cm^−1^) were able to identify both PE subject groups compared to their respective matched normotensive control group with over 80% accuracy. Nuclear magnetic resonance (NMR) spectroscopy later revealed serum levels of arginine and citrate were decreased significantly in early-onset compared to late-onset PE while levels of glutamate, choline, alanine and lactate were significantly increased in the early-onset subject group (80). A preliminary study using Raman spectroscopy of serum for the identification of biomarkers and subsequent diagnosis of PE in a small cohort of women (*n* = 9) at an unknown stage of gestation revealed spectral differences between healthy and preeclamptic women. Spectral differences were attributed to decreasing intensity of amino acid associated bands and increasing band intensities of lipid-associated molecules in PE. Using principle component analysis (PCA) and linear discriminant analysis (LDA), sensitivity and specificity of 78 and 90% respectively was obtained for the diagnosis of PE (81).

**Figure 3:**
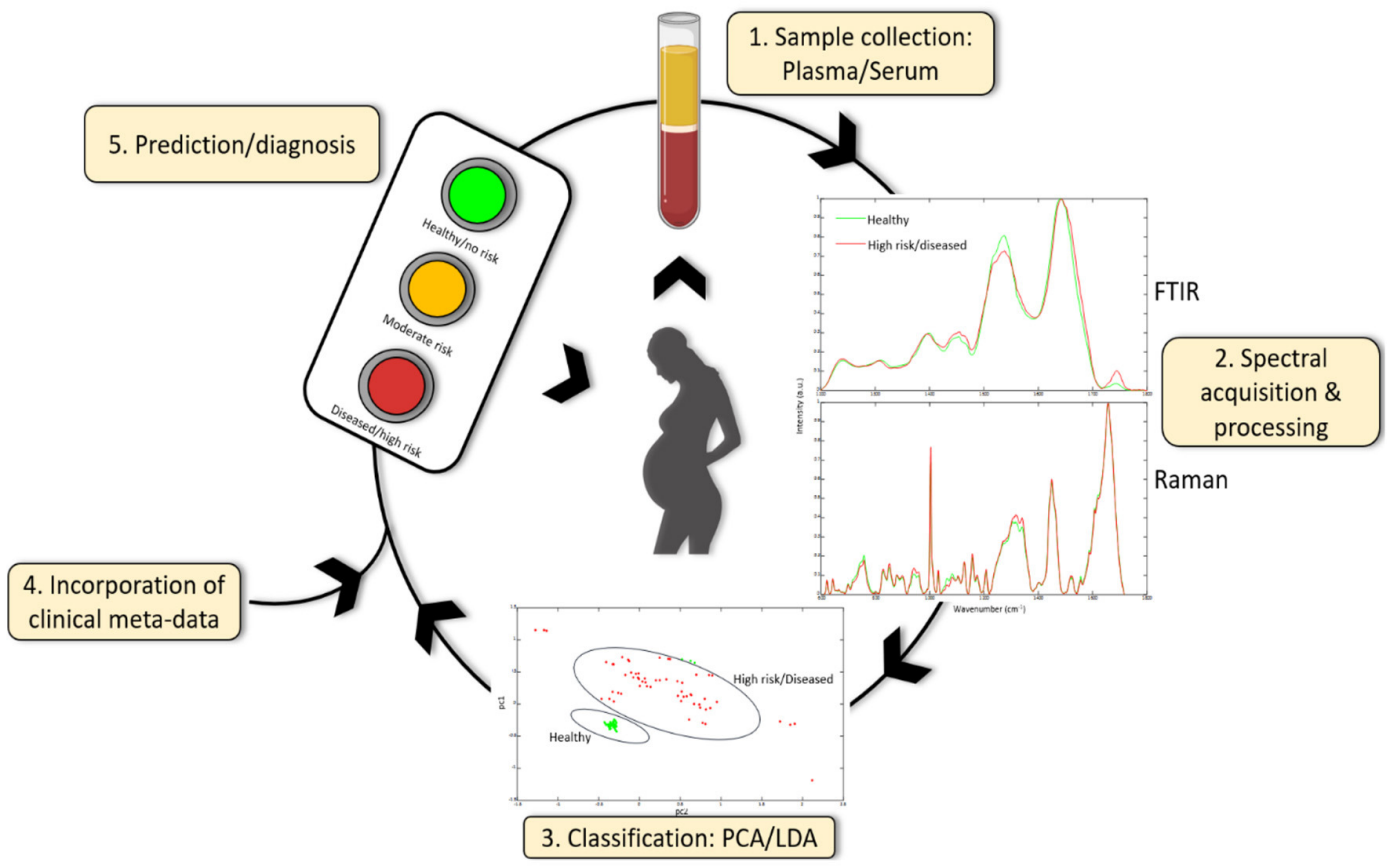
Serum or plasma obtained from pregnant women can be used to acquire spectra using FTIR or Raman spectroscopy. Spectral data can be further classified using chemometric methods (and machine learning) including principle component analysis (PCA) and linear discriminant analysis (LDA) and can be combined with other clinical meta-data to give a diagnosis or prediction of disease development. Additionally, spectral data can identify novel candidate biomarkers which may be useful in future diagnostic test development.

These studies certainly suggest that pursuing the use of vibrational spectroscopy for PE diagnostics and prognostics is worthwhile but larger cohorts of women at early stages of gestation are required. We are of course some way from validating this approach and there is a danger that the same limitations that have beset other PE diagnostic paradigms apply here as well. Nevertheless, the use of robust chemometrics such as PCA and LDA, in conjunction with machine learning and regression modeling, and high-throughput methods will validate the feasibility of vibrational spectroscopy for screening, diagnosing and monitoring PE. An overview of the use of these techniques in obstetric care is illustrated in Figure 3. While the early goal is to better facilitate the diagnosis of preeclampsia and to provide better management of women diagnosed with preeclampsia, parallel efforts are being made to develop this as a predictive tool usable much earlier in pregnant in conjunction with other routine screenings common to pregnancy care. Key will be ensuring this remains a low cost, easy to implement test to encourage uptake in communities where there is a personal cost per test to the pregnant woman.

The application of vibrational spectroscopy in obstetrics is of course not limited to PE. GDM, defined as glucose intolerance or hyperglycemia first recognized during pregnancy, is another pregnancy disorder with a significant health burden. GDM is diagnosed using a glucose tolerance test which carries a significant burden for pregnant women and healthcare staff. Women must fast overnight until the completion of the test the following morning, attend hospital for 2–3 h, consume an unpleasant glucose solution and have 2 sets of blood tests, all of which is undesirable during pregnancy and requires considerable time and effort. Similarly, obstetric cholestasis is another pregnancy disorder that requires extensive and repeat testing while fasting (82). Therefore, alternative diagnostic approaches of which vibrational spectroscopy might be one are sorely needed to reduce the tangible and intangible costs of current GTT-based screening for GDM and bile acid/liver enzyme tests for obstetric cholestasis.

As sample preparation is minimal for both Raman spectroscopy and FTIR and there are no added requirements of specialized reagents for conventional vibrational spectroscopic techniques these methods might be adoptable by low resource countries to improve antenatal monitoring capacity and capability by serving as a point of care (POC) or community-based testing regimes. The considerable interest in developing handheld devices for diagnostic application will further support this. Handheld Raman and FTIR devices have long been used in the pharmaceutical industry and have recently begun to transition into the field of diagnostics. Interoperative Raman probes have been used in real time to aid surgical resection of brain cancer with high sensitivity (93%) and specificity (91%), improving clinical decision making (83). Raman probes have also been utilized *in vivo* to distinguish skin cancer, precancerous and benign skin lesions with superior sensitivity and specificity to that of conventional methods (84). The development of POC vibrational spectroscopy instruments would require the incorporation of microfluidic devices which may be challenging from an engineering perspective. However, such devices have recently been integrated into surface-enhanced Raman spectroscopy platforms for diagnosis of infectious diseases which act to miniaturize the technology making it applicable as a POC testing device (85). As this technology is progressed for obstetric care, inclusion of cohorts in Sub Saharan Africa will be important to ensure that biological and socio-economic conditions found in this region are represented. In SSA burdens from obstetric complications together with infectious and non-communicable diseases all overlap, so it is very likely that there will be unique findings upon vibrational spectroscopy. The high numbers of pathological pregnancies in SSA will enable rapid development of high-performance algorithms (based on clinical-epidemiological risk factors) and tests (based on biomarker identification and validation). This clinical setting also allows evaluation of adverse pregnancy outcomes which are rare in high income cohorts and yet of global health importance, such as stillbirth and severe pre-eclampsia. Indicated preterm delivery, as a result of PE, is major cause of poor neonatal outcomes, especially in low-and-middle income countries where neonatal services are not as well developed. Continued advances in handheld devices and the appropriate biomarker chemometrics developments would offer up vibrational spectroscopy as a viable low-cost predictor tool that could be used in the field and in remote locations, thereby impacting preterm delivery and adverse obstetric outcomes.

## Author Contributions

OR, CJ, and CT contributed to the conception and design of the review. OR wrote first draft of the manuscript. CJ, HG, AN, PD, and CT wrote sections of the manuscript. SJ, MM, TT, JB, AN, and MN provided clinical oversight. OR, CJ, EP, and PD provided oversight into the vibrational spectroscopy techniques. All authors contributed to the article and approved the submitted version.

## Conflict of Interest

CT has funding from Bristol Myers Squibb. The remaining authors declare that the research was conducted in the absence of any commercial or financial relationships that could be construed as a potential conflict of interest.
